# MRI-Derived Fat Fraction Is Superior to Ultrasonography in Predicting Subclinical Atherosclerosis in Metabolically Healthy Non-alcoholic Fatty Liver Disease

**DOI:** 10.7759/cureus.88636

**Published:** 2025-07-23

**Authors:** Sunil Bhawariya, Arshia Bhardwaj, Arshdeep Singh, Riya Sharma, Rajiv Kapoor, Ashutosh Bawa, Liza Joshi, Gopal Bhardwaj

**Affiliations:** 1 General Medicine, General Hospital Sector 6 Panchkula, Panchkula, IND; 2 Gastroenterology, Dayanand Medical College and Hospital, Ludhiana, IND; 3 Research and Development, Dayanand Medical College and Hospital, Ludhiana, IND; 4 Radiology, General Hospital Sector 6 Panchkula, Panchkula, IND; 5 General Surgery, Government Medical College Srinagar, Srinagar, IND; 6 Pathology, General Hospital Sector 6 Panchkula, Panchkula, IND; 7 Internal Medicine, General Hospital Sector 6 Panchkula, Panchkula, IND

**Keywords:** carotid intima-media thickness, hepatic steatosis, metabolically healthy nafld, mri-pdff, subclinical atherosclerosis

## Abstract

Background: Subclinical atherosclerosis in metabolic dysfunction-associated steatotic liver disease (MASLD) is well established. However, its presence in patients with metabolically healthy non-alcoholic fatty liver disease (MH-NAFLD) remains unclear.

Aim: To determine the prevalence of subclinical atherosclerosis in patients with MH-NAFLD and to evaluate whether higher grades of hepatic steatosis are associated with increased carotid intima-media thickness (CIMT).

Methods: In this prospective observational study, adults with ultrasonography (USG)-confirmed hepatic steatosis and no components of metabolic syndrome were compared with age- and sex-matched healthy controls. Hepatic steatosis was quantified using MRI-proton density fat fraction (MRI-PDFF), and subclinical atherosclerosis was assessed via CIMT. A CIMT ≥ 0.84 mm was considered elevated. Associations were evaluated using Spearman’s correlation and hierarchical linear regression.

Results: A total of 100 participants (median age 36.5 years, IQR 29.0-45.25) were enrolled: 50 with MH-NAFLD (28, 56% males) and 50 matched controls (31, 62% males). MRI-PDFF stratified MH-NAFLD patients as Grade 0 (8, 16%), Grade 1 (23, 46%), Grade 2 (11, 22%), and Grade 3 (8, 16%). Elevated CIMT was observed in 13 (26%) MH-NAFLD participants vs 1 (2%) control (p = 0.001). None of the patients with Grade 0 steatosis had elevated CIMT, whereas all with Grade 3 did. MRI-PDFF grading showed a strong correlation with CIMT (ρ = 0.80, p < 0.0001). In hierarchical regression, MRI-PDFF significantly improved CIMT prediction (ΔR² = 0.39, p < 0.001), while USG lost statistical significance.

Conclusion: MH-NAFLD is associated with increased CIMT. MRI-PDFF is a superior predictor of subclinical atherosclerosis compared to USG, highlighting its utility for vascular risk stratification.

## Introduction

Metabolic dysfunction-associated steatotic liver disease (MASLD) has emerged as a significant global health challenge and is currently the leading cause of chronic liver disease worldwide [1]. Its global prevalence is estimated at 30%, while in India, the prevalence has been reported at 38.6% [2-4]. This growing burden is largely driven by the increasing incidence of obesity, type 2 diabetes mellitus (T2DM), and sedentary lifestyles. However, a distinct subset of individuals with MASLD exists, referred to as metabolically healthy non-alcoholic fatty liver disease (MH-NAFLD), who do not exhibit these classical metabolic abnormalities [5,6].

MASLD is now understood not merely as a hepatic condition but as a systemic disease with diverse extrahepatic manifestations. Among these, cardiovascular disease represents the primary cause of morbidity and mortality [7,8]. Insulin resistance and atherogenic dyslipidemia play central roles in the development of premature atherosclerosis in MASLD [9]. Hepatic steatosis, irrespective of liver dysfunction, also contributes to the release of pro-inflammatory cytokines such as TNF-α and IL-6, pro-coagulant factors, and reactive oxygen species. These factors collectively impair endothelial function, promote atherogenesis, and contribute to vascular remodeling, including carotid intima-media thickening (CIMT) [10,11]. CIMT is a well-established, noninvasive surrogate marker of subclinical atherosclerosis. Prior studies have demonstrated that individuals with MASLD have increased CIMT and are at greater risk for cardiovascular events, including coronary artery disease and stroke [12].

Magnetic resonance imaging-based proton density fat fraction (MRI-PDFF) is a highly sensitive and quantitative method for assessing hepatic fat content. It is capable of detecting even mild steatosis, below 5%. It offers superior diagnostic accuracy compared to ultrasonography (USG) and computed tomography because it evaluates fat distribution across the whole liver and minimizes sampling error [13,14].

While prior studies have demonstrated a positive association between MASLD and increased CIMT, most of this evidence has focused on patients with overt metabolic dysfunction. There is limited understanding of whether MH-NAFLD, in the absence of traditional metabolic risk factors, is similarly associated with subclinical atherosclerosis. A deeper understanding of this relationship has important clinical implications because MH-NAFLD is often perceived as a benign entity and may be under-recognized in cardiovascular risk stratification frameworks. The present study aims to evaluate the association between hepatic steatosis, as quantified by MRI-PDFF, and CIMT in patients with MH-NAFLD.

## Materials and methods

Methods

Study Design

This prospective, observational, hospital-based study was conducted between April 2023 and March 2024 at a public healthcare facility in North India. The study was approved by the institutional ethics committee (GCH/EC/2021/1826/(24.010), dated February 3, 2023). The Strengthening the Reporting of Observational Studies in Epidemiology (STROBE) guidelines for reporting observational studies were followed.

Study Population

Adult participants (aged ≥18 years) with radiologically confirmed hepatic steatosis of any grade on abdominal USG were considered eligible for inclusion. The diagnosis of hepatic steatosis was established based on standardized USG criteria, including increased echogenicity of the liver parenchyma relative to the renal cortex, attenuation of the USG beam, and loss of portal vein wall echogenicity [15]. To further characterize and quantify hepatic fat content, all enrolled individuals underwent MRI of the abdomen using a standardized imaging protocol.

Participants were included only if they had isolated hepatic steatosis on USG in the absence of overt metabolic derangements. Individuals with any of the following metabolic risk factors were excluded: (i) increased waist circumference (≥94 cm in males, ≥80 cm in females) or body mass index (BMI) ≥25 kg/m²; (ii) impaired fasting glucose, elevated glycated hemoglobin (HbA1c ≥6.5%), a previously established diagnosis of type 2 diabetes mellitus, or current use of antidiabetic therapy; (iii) dyslipidemia; and (iv) hypertension [16]. Additional exclusion criteria comprised alcohol consumption exceeding 30 grams per day for males or 20 grams per day for females; a diagnosis of chronic liver diseases including viral hepatitis (hepatitis B or C), autoimmune hepatitis, or drug-induced liver injury; and prior or current use of known steatogenic agents within the preceding 12 months, including corticosteroids, amiodarone, methotrexate, tamoxifen, tetracycline, high-dose estrogens, or valproic acid. Participants receiving saroglitazar, vitamin E, betaine, S-adenosylmethionine, ursodeoxycholic acid, silymarin, or pentoxifylline within the preceding three months, and patients with contraindications to MRI, such as claustrophobia, presence of metallic implants, or unwillingness to undergo the imaging procedure, were also excluded.

Healthy Controls

Age- and sex-matched healthy individuals were recruited as controls. These were selected from individuals presenting for routine health check-ups at the same healthcare facility. All controls underwent a comprehensive clinical assessment, including a detailed medical history, physical examination, and relevant laboratory investigations. Participants with any clinical, biochemical, or radiological evidence of liver disease or other comorbid conditions were excluded.

MRI Protocol

MRI examinations were performed using a PHILIPS MULTIVA 1.5 Tesla scanner (Amsterdam, Netherlands). A single experienced radiologist with over five years of experience in abdominal imaging interpreted all MRI scans for PDFF estimation. Intra-observer variability was minimized by performing image interpretation in a standardized manner using vendor-provided software with automated region of interest (ROI) placement in liver segments, avoiding vessels and bile ducts. Hepatic steatosis was quantified using the mDIXON-ALL BH sequence to derive the proton density fat fraction (PDFF). PDFF values were classified into four categories: Grade 0 (<6.4%), Grade 1 (6.4% to <16.3%), Grade 2 (16.3% to <21.7%), and Grade 3 (≥21.7%) [17,18].

USG Abdomen

Ultrasound was performed using a high-resolution B-mode scanner with a curvilinear transducer (3.5-5 MHz). Steatosis was graded semi-quantitatively (Grades 0-3) based on echogenicity, diaphragm visibility, and vascular blurring, in accordance with established criteria.

Carotid Intima-Media Thickness Assessment

Bilateral common carotid artery intima-media thickness was measured using high-resolution B-mode Doppler USG (ESAOTE Eight eXP, linear probe 3-13 MHz, Genoa, Italy). Measurements were taken at the far wall of the distal common carotid artery, 1 cm proximal to the carotid bulb. The mean CIMT was calculated as the average of the left and right values. Participants were stratified into two risk categories based on CIMT measurements: low risk (<0.84 mm) and high risk (≥0.84 mm) [19,20].

Outcomes

The primary outcome of the study was to assess the association between the degree of hepatic steatosis, assessed by USG and MRI-PDFF, and CIMT. Secondary outcomes included the distribution of hepatic fat content based on MRI-PDFF among metabolically healthy individuals, comparison of CIMT values between cases and age- and sex-matched healthy controls, and evaluation of the incremental predictive value of MRI-PDFF over USG in explaining the variance in CIMT.

Statistical Analysis

Descriptive statistics were used to summarize the data. Continuous variables were expressed as median (interquartile range), while categorical variables were presented as frequencies and percentages. The Kolmogorov-Smirnov test was applied to assess the normality of data distribution. Between-group comparisons for continuous variables were performed using the Mann-Whitney U test due to non-parametric data distribution. Categorical variables were compared using the chi-square (χ²) test, with Fisher’s exact test applied when expected frequencies were <5. Correlations between variables were evaluated using Spearman’s rank correlation coefficient (Spearman’s rho). To examine the association between hepatic steatosis and CIMT, a hierarchical linear regression analysis was conducted. The dependent variable in all models was CIMT (continuous), and independent variables were entered in two sequential models to assess the incremental explanatory power of advanced imaging over conventional ultrasound. In Model 1, the ultrasound (USG) grade of hepatic steatosis was entered as the sole predictor. In Model 2, MRI-derived proton density fat fraction (MRI-PDFF) was added to the regression model to determine its independent contribution. The F-change statistic was used to evaluate whether the increase in R² between the two models was statistically significant. The F-statistic for each model was reported to indicate overall model significance. A p-value <0.05 was considered statistically significant. Statistical analyses were conducted using IBM SPSS Statistics for Windows, Version 21 (Released 2012; IBM Corp., Armonk, New York).

## Results

Results

Participant Characteristics

A total of 100 participants were enrolled, comprising 50 with MH-NAFLD (28 males, 56%) and 50 age- and sex-matched healthy controls (31 males, 62%). The median age in the MH-NAFLD group was 36.5 years (IQR 28.75-47.00), comparable to the controls (37.0 years, IQR 29.75-45.25; p = 0.84). The baseline characteristics of the population are depicted in Table 1.

Radiological Assessment

On USG, 17 (34%) participants with MH-NAFLD had Grade 1 steatosis, 26 (52%) had Grade 2, and 7 (14%) had Grade 3. The healthy controls did not have hepatic steatosis on USG. MRI-PDFF classified participants with MH-NAFLD as follows: Grade 0 (<6.4%) in 8 (16%), Grade 1 (6.4% to <16.3%) in 23 (46%), Grade 2 (16.3% to <21.7%) in 11 (22%), and Grade 3 (≥21.7%) in 8 (16%). All healthy controls had MRI-PDFF <6.4%.

Association Between MRI-PDFF and USG

**Figure 1:**
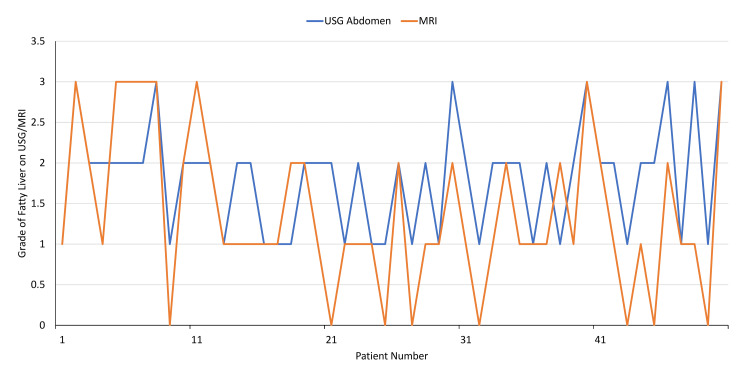
Comparison of hepatic steatosis grading by USG and MRI-PDFF. The line diagram demonstrates the variability in steatosis grading between the two modalities. USG: ultrasonography, MRI-PDFF: magnetic resonance imaging–proton density fat fraction.

A significant association was observed between MRI-PDFF and ultrasonographic grades of steatosis among participants with MH-NAFLD (χ² = 19.36, p = 0.004). MRI-PDFF provided a broader quantitative stratification of hepatic fat, including the detection of extreme (Grade 0 and Grade 3) grades of steatosis not captured by USG (Figure 1).

Carotid Intima-Media Thickness

Mean CIMT was higher in participants with MH-NAFLD (0.69 ± 0.15 mm) compared to controls (0.64 ± 0.07 mm) (p = 0.07). Elevated CIMT (≥0.84 mm) was observed in 13 (26.0%) participants with MH-NAFLD compared to 1 (2.0%) participant in the healthy control group (p = 0.001).

Association Between CIMT and MRI-PDFF

Among participants with MH-NAFLD, none of those with an MRI-PDFF <6.4% (Grade 0, n = 8) demonstrated elevated CIMT. In those with Grade 1 steatosis (n = 23), 22 participants (95.7%) had normal CIMT values. Elevated CIMT was observed in 4 out of 11 participants (36.4%) with Grade 2 steatosis. Notably, all participants with Grade 3 steatosis (n = 8) exhibited elevated CIMT. Among healthy controls, all participants had MRI-PDFF <6.4%, with only one individual presenting with elevated CIMT.

**Figure 2:**
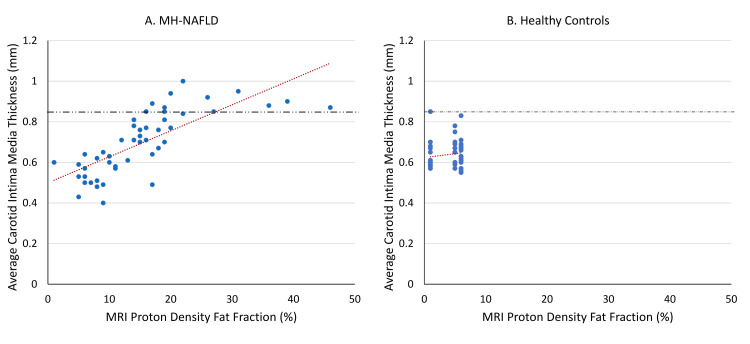
Correlation between hepatic steatosis and CIMT in patients with MH-NAFLD and healthy controls. (A) Strong positive correlation was observed between MRI-PDFF and CIMT in patients with MH-NAFLD (Spearman’s ρ = 0.80, p < 0.0001), with linear regression showing an R² of 0.54. (B) No significant association was seen between MRI-PDFF and CIMT in healthy controls (R² = 0.02). The dotted line represents the regression line; the dashed line indicates the CIMT threshold of 0.84 mm. CIMT: carotid intima-media thickness, MH-NAFLD: metabolically healthy non-alcoholic fatty liver disease, MRI-PDFF: magnetic resonance imaging–proton density fat fraction.

In participants with MH-NAFLD, a very strong positive correlation was observed between MRI-PDFF and CIMT (Spearman’s rho = 0.80, p < 0.0001) (Figure 2).

**Figure 3:**
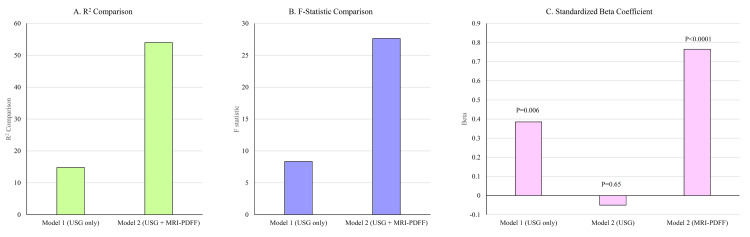
Comparison of regression models evaluating the association between hepatic steatosis and CIMT in participants with MH-NAFLD. (A) Model 2 (USG + MRI-PDFF) demonstrated a higher explanatory power compared to Model 1. (B) The F-statistic was higher in Model 2 than in Model 1. (C) In Model 1, USG grading was a significant predictor, but its effect diminished in Model 2, while MRI-PDFF emerged as a robust independent predictor of CIMT. USG: ultrasonography, MRI-PDFF: magnetic resonance imaging–proton density fat fraction, CIMT: carotid intima–media thickness, MH-NAFLD: metabolically healthy non-alcoholic fatty liver disease.

To investigate the association between hepatic steatosis and CIMT, hierarchical regression analysis was performed. In Model 1, USG grade of hepatic steatosis was included as a predictor. The model was statistically significant, explaining 14.8% of the variance in CIMT (R² = 0.148, F(1, 48) = 8.35, p = 0.006). The presence of hepatic steatosis on USG was a significant predictor of CIMT (β = 0.385, t = 2.890). In Model 2, MRI-derived fat fraction was added to the model. The inclusion of this variable led to a significant increase in explained variance (ΔR² = 0.39), with the overall model explaining 54% of the variance in carotid intima thickness (R² = 0.540, F(2, 47) = 27.63, p < 0.001). In this model, MRI-PDFF emerged as a significant independent predictor (β = 0.765, t = 6.334, p < 0.001), while the USG grade of hepatic steatosis was no longer statistically significant (p = 0.66) (Figure 3).

**Table 1:** Baseline Characteristics The values are provided as median (interquartile range) or n (%) as applicable. USG: ultrasonography, MRI-PDFF: magnetic resonance imaging–based proton density fat fraction.

Variables	NAFLD (n=50)	Healthy Controls (n=50)	Significance
Age (years)	36.50 (28.75-47)	37 (29.75-45.25)	0.84
Male ((n (%))	28 (56)	31 (62)	0.54
Body mass index (kg/m^2^)	23.88 (23.04-24.59)	23.36 (22.70-24.34)	0.42
Height (m)	1.65 (1.60-1.72)	1.64(1.59-1.68)	0.84
Weight (kg)	71 (68-74.25)	65 (62-68)	0.22
Fasting blood sugar (mg/dL)	98 (89-104)	94 (79-101.25)	0.07
High-density lipoprotein cholesterol (mg/dL)	38.50 (36-42)	41 (38-44)	1.00
Triglycerides (mg/dL)	40 (38-43)	41 (38-44)	1.00
Total cholesterol (mg/dL)	196 (182.75-207.25)	194.50 (183-206.50)	0.84
Aspartate aminotransferase	37 (33-41.25)	37 (32.75-42)	1.00
Alanine aminotransferase	36 (32.75-41.25)	36 (32-41)	0.84
Grade of hepatic steatosis on USG abdomen			
Grade 1	17 (34)	Nil	
Grade 2	26 (52)	Nil	
Grade 3	7 (14)	Nil	
MRI-PDFF hepatic fat fraction			
Grade 0	8 (16)	Nil	
Grade 1	23 (46)	Nil	
Grade 2	11 (22)	Nil	
Grade 3	8 (16)	Nil	

## Discussion

This prospective observational study aimed to provide insights into the prevalence of subclinical atherosclerosis and the association between hepatic steatosis, as quantified by MRI-PDFF, and subclinical atherosclerosis in individuals with MH-NAFLD. Our findings reinforce the growing recognition of MASLD as a multisystem disorder with significant cardiovascular implications.

Elevated CIMT (≥0.84 mm) was observed in 26% of patients with MH-NAFLD compared to only 2% of controls, suggesting that hepatic steatosis alone may contribute to early vascular changes, independent of classical metabolic risk factors. While USG remains a widely used and accessible imaging modality, MRI-PDFF detected Grade 3 steatosis in a subset of patients not well delineated by USG, highlighting the superior sensitivity of MRI-PDFF in the quantitative stratification of hepatic steatosis. Importantly, CIMT increased progressively with higher grades of steatosis on both USG and MRI-PDFF. A strong correlation was observed between MRI-PDFF and CIMT (Spearman’s ρ = 0.80, p < 0.0001).

Our findings highlight two key insights. First, both imaging modalities (USG and MRI-PDFF) identified associations between hepatic steatosis and subclinical atherosclerosis, reinforcing the liver-vascular axis in metabolic health [12,21,22]. Second, and more critically, the quantitative precision of MRI-PDFF appears to capture the severity of hepatic steatosis more accurately, translating into a significantly stronger predictive value for vascular risk. The large increase in R² (ΔR² = 0.39) and high F-change statistic (ΔF = 19.28, p < 0.001) indicate that MRI-PDFF adds substantial explanatory power beyond what is captured by conventional USG grading. The diminished significance of USG in the combined model may be due to its limited sensitivity and operator dependency, which can lead to misclassification or underestimation of hepatic fat content. In contrast, MRI-PDFF offers a standardized, reproducible, and highly sensitive assessment of hepatic steatosis, capable of detecting subtle variations that may correlate with vascular endothelial dysfunction and arterial remodeling [14].

This study is among the few to specifically evaluate the relationship between hepatic steatosis and subclinical atherosclerosis in a well-defined cohort of individuals with MH-NAFLD, explicitly excluding those with classical metabolic syndrome components such as obesity, diabetes, hypertension, and dyslipidemia. By focusing on this unique population, the study isolates hepatic steatosis as an independent contributor to early vascular changes, as measured by CIMT. This has important clinical implications, suggesting that cardiovascular risk in MASLD may be under-recognized in patients lacking traditional risk factors [23,24]. We also found that healthy controls with MRI-PDFF <6.4% had a very low prevalence of elevated CIMT (2%), further supporting the specificity of hepatic steatosis as a determinant of subclinical atherosclerosis in this context.

Key strengths of our study include the use of MRI-PDFF for quantitative assessment of hepatic steatosis, exclusion of confounding metabolic comorbidities, and standardized evaluation of CIMT using high-resolution Doppler USG. However, certain limitations must be acknowledged. First, the study’s cross-sectional design precludes causal inference between hepatic steatosis and atherosclerosis. Second, the sample size, while adequate for detecting associations, limits the generalizability of subgroup analyses. Third, the use of a single-center hospital-based cohort may introduce selection bias. Lastly, we did not assess systemic inflammatory markers or other atherosclerosis-related parameters, which could have provided additional mechanistic insights.

## Conclusions

Our study demonstrates a strong positive correlation between hepatic steatosis, as measured by MRI-PDFF, and CIMT in patients with MH-NAFLD, independent of classical metabolic risk factors. MRI-PDFF appears to outperform conventional USG in identifying individuals with hepatic steatosis who are at increased risk of subclinical atherosclerosis. These findings support the integration of MRI-PDFF into cardiovascular risk stratification frameworks for patients with MH-NAFLD, potentially guiding early preventive interventions in this high-risk population. Prospective longitudinal studies are warranted to validate these findings and to explore the impact of hepatic fat reduction on cardiovascular outcomes.
